# Cellular Entry of the Diphtheria Toxin Does Not Require the Formation of the Open-Channel State by Its Translocation Domain

**DOI:** 10.3390/toxins9100299

**Published:** 2017-09-22

**Authors:** Alexey S. Ladokhin, Mauricio Vargas-Uribe, Mykola V. Rodnin, Chiranjib Ghatak, Onkar Sharma

**Affiliations:** 1 Department of Biochemistry and Molecular Biology, University of Kansas Medical Center, Kansas City, KS 66160, USA; mauricio.vargasu@gmail.com (M.V.-U.); mrodnin@kumc.edu (M.V.R.); c.ghatak79@gmail.com (C.G.); 2 Department of Microbiology and Immunobiology, Harvard Medical School, Boston, MA 02115, USA; Onkar_Sharma@hms.harvard.edu

**Keywords:** membrane translocation, pH-dependent refolding, bilayer insertion, tryptophan fluorescence, depth-dependent fluorescence quenching

## Abstract

Cellular entry of diphtheria toxin is a multistage process involving receptor targeting, endocytosis, and translocation of the catalytic domain across the endosomal membrane into the cytosol. The latter is ensured by the translocation (T) domain of the toxin, capable of undergoing conformational refolding and membrane insertion in response to the acidification of the endosomal environment. While numerous now classical studies have demonstrated the formation of an ion-conducting conformation—the Open-Channel State (OCS)—as the final step of the refolding pathway, it remains unclear whether this channel constitutes an in vivo translocation pathway or is a byproduct of the translocation. To address this question, we measure functional activity of known OCS-blocking mutants with H-to-Q replacements of *C*-terminal histidines of the *T*-domain. We also test the ability of these mutants to translocate their own *N*-terminus across lipid bilayers of model vesicles. The results of both experiments indicate that translocation activity does not correlate with previously published OCS activity. Finally, we determined the topology of TH5 helix in membrane-inserted *T*-domain using W281 fluorescence and its depth-dependent quenching by brominated lipids. Our results indicate that while TH5 becomes a transbilayer helix in a wild-type protein, it fails to insert in the case of the OCS-blocking mutant H322Q. We conclude that the formation of the OCS is not necessary for the functional translocation by the *T*-domain, at least in the histidine-replacement mutants, suggesting that the OCS is unlikely to constitute a translocation pathway for the cellular entry of diphtheria toxin in vivo.

## 1. Introduction

The diphtheria toxin enters and kills the cell through the combined action of its three domains: the catalytic domain (*C*-domain), the translocation domain (*T*-domain), and the receptor-binding domain (*R*-domain) [1]. The entry process starts with the *R*-domain recognizing the HB-EGF receptor on the host cell plasma membrane, leading to the endocytosis of the entire receptor-toxin complex. Subsequent acidification of the endosomal compartment induces the refolding of the *T*-domain and its insertion into the lipid bilayer. The latter causes the translocation of the *C*-domain across the endosomal membrane, which finally inhibits protein synthesis in the cytosol. Deciphering the missing molecular details of diphtheria toxin’s cellular entry is relevant for understanding the entry of other toxins [2] and for the development of biomedical applications of targeted drug delivery. Indeed, diphtheria toxin has already been utilized as a prospective anti-cancer agent for the targeted delivery of cytotoxic therapy to cancer cells [3,4,5,6,7,8,9,10,11,12,13,14,15,16]. Normally, the targeted delivery is achieved by deleting the cell receptor-binding *R*-domain and combining the remaining portion (containing *T*- and *C*-domains) with proteins that selectively bind to the surface of cancer cells. Even a “receptorless” toxin (i.e., without the *R*-domain) is cytotoxic to a variety of cancer cell lines [3]. Because cancerous cells are known to produce a slightly acidic extracellular environment, we pursue the idea that targeting of “receptorless” toxin is assured by pH-triggered membrane insertion of the *T*-domain similar to that of the *pH*-*Low Insertion Peptide* (pHLIP) [17,18,19,20,21,22,23,24,25,26]. The previously demonstrated ability of the isolated *T*-domain to translocate relatively large macromolecules [27] in a pH-dependent manner makes it a potential candidate for targeting tumors.

**Figure 1:**
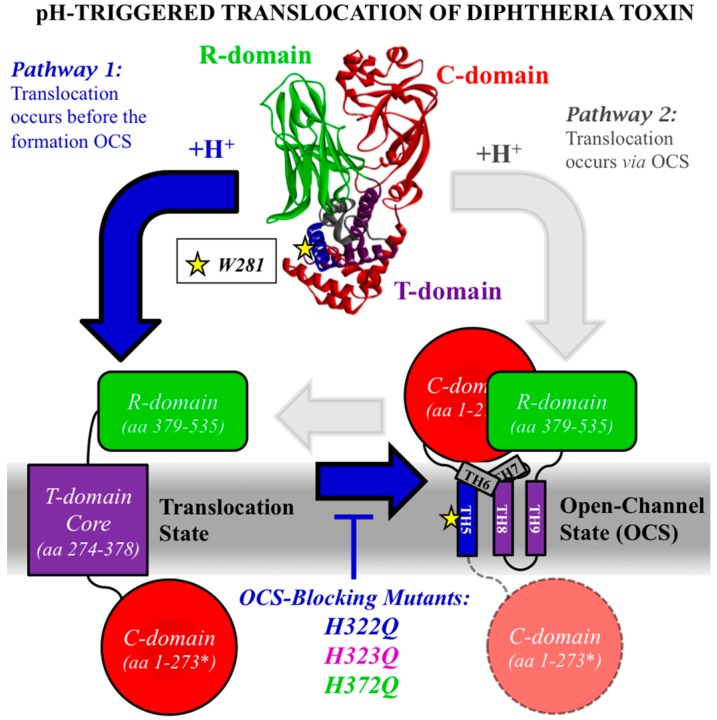
Schematic representation of two possible pathways for the translocation of the catalytic (C) domain across the lipid bilayer and formation of the Open-Channel State (OCS) by the translocation (T) domain of the diphtheria toxin. The starting structure on top corresponds to the crystal structure of the toxin at neutral pH [42], and consists of the *C*-domain (red), *T*-domain (helices color-coded according to OCS topology), and *R*-domain (green). The yellow star represents the position of W281 within the lipid bilayer in the OCS, which was used in this study to monitor the formation of the OCS (Figure 2 and Figure 3). In pathway 1 (blue arrows), diphtheria toxin first translocates the *C*-domain and the *T*-domain’s *N*-terminus across the lipid bilayer by an unknown mechanism (bottom left cartoon). The formation of the Open-Channel State (bottom right cartoon) is a consequence of the translocation step. In pathway 2 (gray arrows), the *T*-domain first adopts the OCS conformation, which then serves as passageway for the translocation of the *C*-domain and the *T*-domain’s *N*-terminal helices. The topology of the *T*-domain in the OCS, i.e., transmembrane helices TH5 (blue helix), and TH8-TH9 (purple helical hairpin), and interfacial helices TH6 and TH7 (gray helices), is based on conductance measurements on planar bilayers [39,40,43] and supported by spectroscopic data in lipid vesicles [30,44]. Replacement of the *C*-terminal histidines of the *T*-domain (H322Q, H323Q, H372Q) are known to strongly reduce formation of channels in planar bilayers by blocking the formation of the OCS conformation [37,38]. Because these replacements do not affect the translocation of the *N*-terminus of the isolated *T*-domain in vitro (Figure 4a), nor delivery of the catalytic domain in vivo (Figure 4b), we conclude that the formation of the OCS is not necessary for cellular entry of the diphtheria toxin, which is occurring via Pathway 1.

In recent years, substantial progress has been made in characterizing structural, thermodynamic, and kinetic aspects of diphtheria toxin *T*-domain’s interactions with lipid bilayers. As a result of these studies (Reviewed in [28]), we now understand that the insertion/refolding process occurs along a complex pathway, which includes multiple intermediates with staggered pH-dependent transitions [29,30,31,32,33]. A number of critical titratable residues involved in acid-induced conformational switching have been identified in a series of spectroscopic studies in vitro (e.g., H223 and H257 [32,33,34,35], E349 and D352 [36], and the *C*-terminal histidines H322, H323, and H372 [35,37,38]). Two recent studies of *T*-domain conductivity in planar lipid bilayers provided better understanding of the topological arrangements of the *T*-domain at the late stages of the pathway, at the so-called Open-Channel State (OCS) [39,40]. However, the central puzzle of the *T*-domain action, namely the exact mechanism of translocation, remains unresolved. One of the key aspects of the puzzle was formulated over 30 years ago by Donovan et al. [41]: is the OCS of the *T*-domain a translocation passageway (illustrated in Figure 1 by Pathway 2) or a byproduct of translocation (Pathway 1)? In this study, we present functional and spectroscopic evidence that suggest the latter option as the preferred one.

## 2. Results and Discussion

### 2.1. Comparing the Two Translocation Pathways

We illustrate two alternative pathways of translocation of the catalytic domain of diphtheria by the translocation domain in the scheme in Figure 1. The starting structure at the top is the crystallographic structure of the toxin at neutral pH [42]. The two cartoons at the bottom represent the arrangement of the toxin upon the insertion of the *T*-domain into the lipid bilayer at acidic pH, with the *R*-domain remaining on one side of the membrane (green shape) and catalytic domain translocated across the bilayer along with the *N*-terminal helices TH1-4 of the *T*-domain (red circle, residues 1–273). On the right, the hydrophobic core of the *T*-domain is drawn in the OCS conformation with the three transmembrane helices, TH5, TH8, and TH9, and a dipped hairpin formed by shorter helices TH6 and TH7. This topology is based on a combination of measurements of channel activity in planar bilayers [39,40,43] and is supported by additional spectroscopic evidence in model lipid vesicles ([30,44] and Kyrychenko et al., Journal of Membrane Biology, in press). The translocation Pathway 2 suggests that OCS is a functionally relevant state, which provides a passageway for translocation of the presumably unfolded catalytic domain to the other side of the bilayer (red circle—before translocation; pink circle—after translocation). In contrast, Pathway 1 indicates that the OCS is formed after the translocation, and passageway through the bilayer is formed via an unknown, possibly transient conformation. In order to distinguish between the two pathways, we will use new and published data to compare how various mutations affect the following measures of *T*-domain activity: (a) conductance in planar bilayers (i.e., formation of the OCS) [37,38,45,46,47,48], (b) *N*-terminus translocation in vesicles [37,49], and ultimately (c) cell death assay based on inhibition of protein synthesis in vivo [37,46,48,50].

### 2.2. Spectroscopic Evidence for the Difference in TH5 Topology in WT and in OCS-Blocking Mutant H322Q

**Figure 2:**
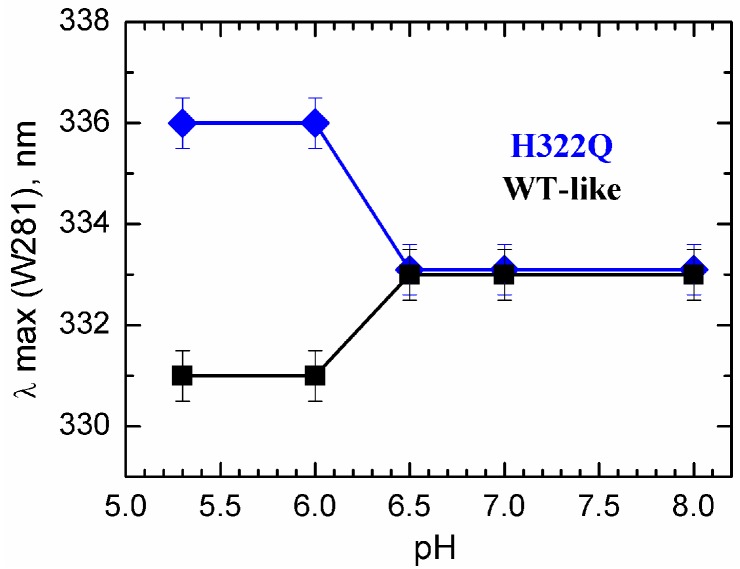
pH-triggered formation of the OCS conformation studied by tryptophan fluorescence. Transmembrane insertion of TH5 is monitored by measuring the position of maximum of emission (λ_max_) of W281 in mutants of the *T*-domain carrying the W206Y replacement. The WT-like protein (black) and the mutant H322Q (blue) show different spectral behavior upon acidification of the medium in the presence of lipid vesicles. Blue-shift in the case of the WT-like protein suggests transbilayer insertion of TH5, while red-shift in the case of the mutant H322Q indicates an alternative topology of this helix.

We used tryptophan fluorescence to explore the effect of the *C*-terminal histidine replacement in H322Q mutant in the pH-triggered conversion of the *T*-domain into an OCS-like conformation in model lipid vesicles. The transmembrane positioning of TH5 helix is a hallmark of the OCS structure [40,43], making W281 located in its middle a convenient spectroscopic probe for membrane insertion. Previously, we have (a) reported that replacement of *C*-terminal histidines (especially H322) has a strong inhibitory effect on OCS activity and (b) shown a correlation between the tryptophan emission signal in vesicles and formation of the OCS in planar bilayers [38]. The reported spectral red-shift observed with OCS-blocking mutants is consistent with lack of insertion of TH5. However, the presence of two tryptophans complicates the interpretation. Now, we reexamine the spectral properties of the *T*-domain in the context of the single W281 by replacing the other tryptophan residue, W206, with a tyrosine in both the WT *T*-domain (from now on referred to as WT-like protein) and the H322Q OCS-blocking mutant. Our data in Figure 2 indicate that in the folded soluble state, at neutral pH, W281 is in an identical environment in both proteins. Lowering the pH below 6.5 in the presence of lipid vesicles results in a very different behavior. While in the WT-like protein, the emission maximum shifts toward shorter wavelengths, consistent with membrane insertion; in H322Q, the shift is toward longer wavelengths. The resulting 5 nm difference in position of fluorescence maximum indicates a much more hydrophobic environment of W281 in the native protein compared to that in H322Q, supporting a difference in membrane topology of TH5 helix in both proteins.

We emphasize that because of the very nature of fluorescence methodology, there can be no one-to-one link between spectral position and structure. Our data prove that known OCS-blocking mutation also results in alteration of bilayer insertion of TH5, reported by environment-sensitive shift of W281 fluorescence. Previously, we reported similar shifts for the WT protein [38], but in that case, it was impossible to specifically assign those to TH5 because of the presence of second fluorophore, W206. The results obtained with W206Y mutant (Figure 2) confirm our previous conclusion that OCS-blocking mutation H322Q alters bilayer insertion of TH5. 

**Figure 3:**
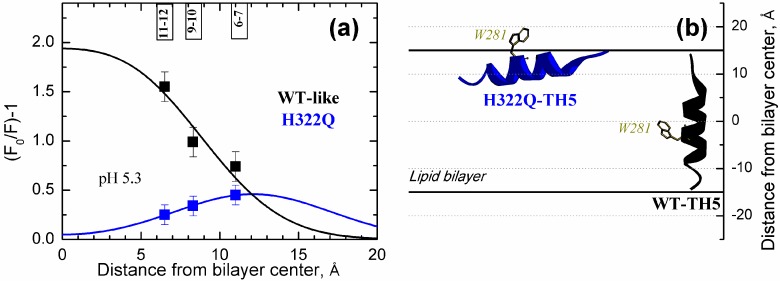
Depth-dependent fluorescence quenching measurements of W281 in WT-like (black) and H322Q *T*-domain (blue) using brominated lipids (both *T*-domain constructs carry W206Y mutation). (**a**) Quantitative analysis of penetration of W281 into the lipid vesicles containing brominated lipids. Relative quenching efficiency, F_0_/F-1 (where F-fluorescence measured in the presence of bromolipids and F_0_-fluorescence without bromolipids) is plotted against the average distances of bromine atoms from the center of the bilayer (11.0 Å, 8.3 Å, and 6.5 Å for 6-7-dibromo-PC, 9-10-dibromo-PC, and 11-12-dibromo-PC, respectively [54]). Solid lines represent results of fitting with a simplified version of Distribution Analysis (Equation (1)) with the following parameters: *h_m_* = 4.5 ± 1.5 Å, S = 1.5 ± 0.3, and *δ* = 5.0 Å(fixed) for WT-like, and *h_m_* = 12.1 ± 0.1 Å, S = 0.46 ± 0.1, and *δ* = 5.0 Å(fixed) for H322Q. (**b**) Schematic illustration of the proposed topology of TH5 helix in the lipid bilayer for *T*-domain WT and H322Q, consistent with positioning W281 in accordance with the data in panel (**a**). The TH5 adopts transbilayer topology in the OCS conformation for the WT *T*-domain, and interfacial topology for the OCS-blocking H332Q mutant.

To further investigate membrane penetration of W281, we used depth-dependent fluorescence quenching experiments [51,52,53]. Specifically, we measured fluorescence intensity of the membrane-inserted *T*-domain in the absence and presence of bromine atoms attached to specific sites of the lipid acyl chains. In this series, we used all three commercially available bromolipids, each with two Br atoms attached either at positions 6 and 7, 9 and 10, or 11 and 12, and plotted the quenching efficiency versus the independently determined average depth of the atoms in the bilayer [54] (Figure 3). The higher the quenching efficiency with a particular bromolipid, the closer is the transverse position of the fluorophore (W281) to that of the quencher (Br). Our data clearly indicate that the rank order of quenching efficiency of the W281 is different for the two proteins: the deeper the quencher the stronger the quenching for the WT-like protein (black squares) and the shallower the quencher the higher the quenching efficiency for H322Q mutant (blue squares).

For quantitative analysis of the data, we applied a Distribution Analysis (DA) method, which describes the quenching profile with a sum of two symmetrical Gaussian functions, representing cis-leaflet and trans-leaflet quenching [52,55]. Generally, DA uses three independent fitting parameters, one for the average depth of the fluorophore (*h_m_*), one for the width of the fluorophore’s transverse distribution (*δ*), and one for quenching efficiency (S), related to fluorophore‘s exposure to the lipid phase [56]. In this case, because the profiles are poorly resolved (i.e., maxima lie outside of the range of depths probed by quenchers), we had to use only two and fixed the *δ* to the value of 5.0 Å, roughly corresponding to the average value for such experiments. This is a commonly used simplifying approach [52,57], and the small variation of the value of *δ* does not change the overall result but affects only the quality of the fit (not shown). We estimated average depths of 4.5 ± 1.5 Å and 12.1 ± 0.1 Å from the center of the bilayer for the WT-like protein and H322Q, respectively. These values indicate a deep membrane penetration of W281 in the case of the WT-like protein and a shallow location of the same residue for the H322Q mutant. The suggested topology of TH5 in both cases, also supported by position of maximum emission (Figure 2), is drawn in Figure 3b, with TH5 as a transbilayer segment for the WT-like toxin and an interfacial helix for the mutant. We suggest that the mutation H322Q impairs the proper insertion of TH5, which is needed for the formation of the OCS.

### 2.3. Translocation Activity of OCS-Blocking Mutants of the T-Domain

**Figure 4:**
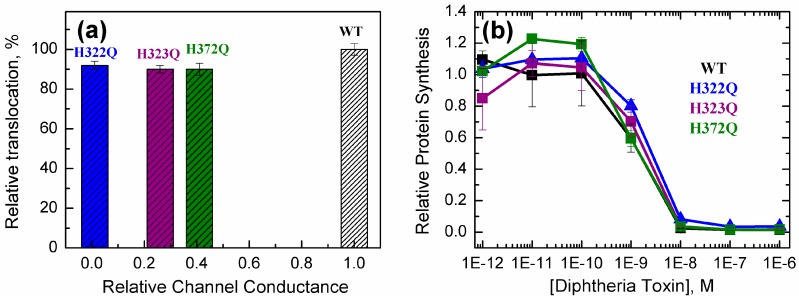
Activity of *T*-domain WT and mutants with single replacements in the *C*-terminal histidines. (**a**) *N*-terminus translocation in mutants of the *T*-domain relative to *N*-terminus translocation of the WT protein at pH 5.8. The assay is based on proteolytic cleavage of *N*-terminal segment preloaded into lipid vesicles followed by SDS-PAGE [49]. The data are normalized to the WT and are plotted against previously published OCS activity [38]. (**b**) Inhibition of protein synthesis by the full-length diphtheria toxin WT and the indicated mutants in CHO-K1 cells after 24 h of intoxication. Protein synthesis measurements were performed as previously detailed [37,58].

We examined the ability of the various mutants of the *T*-domain to bridge the lipid bilayer using a cleavage-based assay developed previously [49]. Briefly, lipid vesicles are preloaded with thrombin, and then a *T*-domain containing a 17-residue thrombin-cleavable tag at the *N*-terminus is added to the external compartment. If *N*-terminus’s translocation occurs upon acidification, the thrombin-cleavable tag enters the vesicle and is cleaved by thrombin. Because this changes the electrophoretic mobility of the tagged *T*-domain, *N*-terminus translocation can be detected and quantified through SDS-PAGE. In Figure 4a we show the relative translocation of the *T*-domain WT and various *C*-terminal single mutants at pH 5.8 plotted against previously determined OCS activity of the same mutants [38] (in order to allow a direct comparison with already published results, all proteins used in Figure 4 contain native W206). The measurements show that the three mutants maintain over 90% of the translocation activity despite the loss of channel activity, suggesting that replacing the OCS-blocking mutations does not alter *N*-terminus’s translocation.

Finally, we have examined the ability of the *T*-domain mutants with His replacements to translocate the catalytic domain across the membrane using a protein synthesis inhibition assay [37,58]. Briefly, we intoxicated plated CHO-K1 cells with serial dilutions of full-length diphtheria toxin, WT or mutant and then monitored protein synthesis by measuring incorporation of L-[4,5-^3^H]-leucine into cells after 24 h of intoxication. In Figure 4b, we show a representative result of a dose-dependent inhibition of protein synthesis by diphtheria toxin WT and the *T*-domain’s mutants with *C*-terminal histidine replacements. The data indicate that both the WT and the mutants completely inhibit incorporation of radiolabeled leucine into cells and do so with similar potency. Because translocation of the *C*-domain is required for inhibition of protein synthesis, this result indirectly shows that replacing these histidines with glutamines does not affect the ability of the *T*-domain to translocate the *C*-domain. Together with the translocation assay (Figure 4a), our data demonstrate that the *T*-domain is capable of translocating its own *N*-terminus and the attached catalytic domain regardless of the OCS formation.

### 2.4. OCS: Critical Intermediate or Byproduct of Translocation

**Figure 5:**
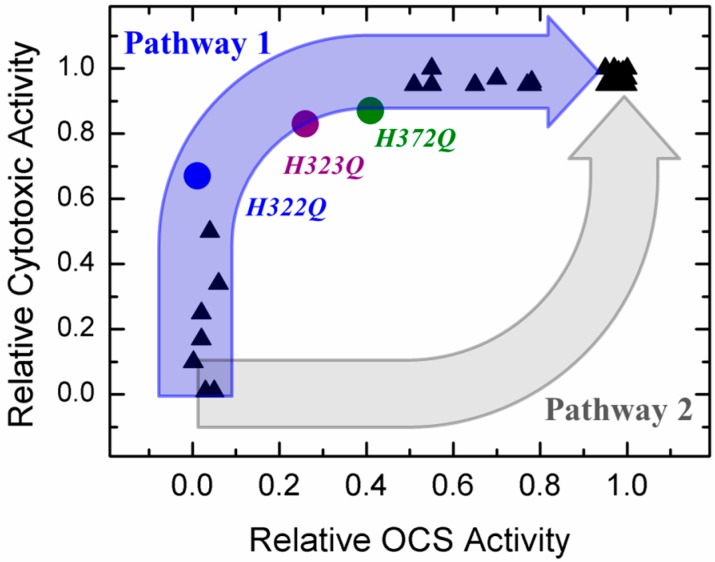
Comparison of the relative cellular toxicity in various mutants of diphtheria toxin and relative OCS activity of the *T*-domain with the same mutations in planar bilayers. Black triangle represents a single set of activity measurements (protein synthesis inhibition in living cells and conductance in planar bilayers) for a mutant reported in references [37,46,48]. The data for H322Q, H323Q, and H372Q mutants (color-coded circles) are from Figure 4b and reference [38]. Blue and gray arrows indicate the expected correlation for the translocation occurring via Pathway 1 or 2, respectively (see Figure 1 and text for details).

We answer the question in the title of this section by comparing the in vitro OCS activity of the *T*-domain in planar bilayers and cytotoxic activity in vivo for a series of mutants of diphtheria toxin with substitutions in the *T*-domain (Figure 5). The data are taken from the literature [37,38,46,48] and from Figure 4b for H322Q, H323Q, and H372Q mutants. The relative cytotoxic activity is defined as a ratio of the concentration of mutant toxin causing half-inhibition of protein synthesis (e.g., data in Figure 4b) to that for the WT toxin. Obviously, if certain mutations have little effect on either in vivo or in vitro activity, the data will cluster close to the point with coordinates 1; 1, corresponding to the WT. In contrast, if both activities are strongly disrupted, the data should cluster around the 0; 0 point. Indeed, many mutants fall into these two categories. The most interesting mutants are those that exhibit intermediate behavior, as they allow one to differentiate the limiting aspect of translocation and distinguish the two pathways outlined in Figure 1. If the formation of OCS constituted the critical intermediate step of the pathway (i.e., pathway 2), one would expect the data to follow a somewhat concave correlation pattern, illustrated by the grey arrow in Figure 5. In reality, the data clearly follow a strongly convex pattern consistent with pathway 1. In the latter pathway, the OCS is a byproduct of translocation that is or is not formed after the translocation occurs. This view is strongly supported by the high WT-like activity of OCS-blocking mutants H322Q, H323Q, and H372Q observed in both in vitro *N*-terminus translocation assay (Figure 4a) and in vivo C-domain translocation assay, required for cytotoxicity (Figure 4b).

## 3. Conclusions and Perspectives

The data presented here clearly demonstrate the ability of diphtheria toxin with OCS-blocking mutations (e.g., H322Q, H323Q, and H372Q [38]) to ensure efficient translocation of its catalytic moiety into the cell (Figure 4b). These mutants are critical in establishing the correlation pattern between in vitro and in vivo activities of toxin mutants (Figure 5), which strongly favors Pathway 1 of cellular entry (Figure 1), in which the OCS is a byproduct of translocation rather than translocation intermediate. These results raise several questions:

1. Is it possible that these mutants take advantage, for some reason, of an entry pathway alternative to that of the WT toxin? While such an option is possible, it seems rather unlikely, because these mutants also appear active in a simplified in vitro translocation assay performed in a reductionist system of artificial lipid vesicles without a transbilayer electrical potential (Figure 4a);
2. Is it possible that the number of molecules in the OCS conformation is only a minor fraction of the entire population in model experiments? Our spectroscopic data indicate otherwise, suggesting a clear correlation between the ability of helix TH5 to insert in the OCS conformation or its precursor even in the absence of transbilayer potential (Figure 2 and Figure 3 and [38]);
3. What is the mechanism of the translocation? Clearly more model and cellular studies will be necessary to fully answer this question. One possibility may involve the formation of a transient passageway due to the perturbation caused by the *T*-domain refolding on bilayer interface. Deciphering the molecular mechanism of this enigmatic system is especially important in light of the potential use for diphtheria toxin *T*-domain as a molecular vehicle for targeted drug delivery.

## 4. Materials and Methods

Materials: Palmitoyl-oleoyl-phosphatidylcholine (POPC) and Palmitoyl-oleoyl-phosphatidyl-glycerol (POPG), 6-7-dibromo-PC, 9-10-dibromo-PC, 11-12-dibromo-PC were obtained from Avanti Polar Lipids (Alabaster, AL, USA).

*T*-domain expression and purification: The diphtheria toxin *T*-domain (amino acids 202–378) was cloned into the Ndel- and EcoRl-treated pET15b vector. The mutations were introduced using Site-Directed Mutagenesis Kit (Stratagene, Santa Clara, CA, USA) according to manufacturer recommendations. WT *T*-domain and the mutants were expressed and purified as described previously [37,38]. Briefly, *E. coli* BL23DELysS cells previously transformed with plasmid carrying T domain WT or mutant were grown in LB medium to OD_600_ = 0.6. Protein expression was induced by addition of 0.8 mM IPTG and grown at 24 °C for 16 h. Cells were cleared by centrifugation, and pellets were resuspended in lysis buffer (25 mM Tris-HCl, 300 mM NaCl, 5 mM imidazole, lysozyme 0.1 mg/mL (Fisher Scientific, Pittsburgh, PA, USA), protease inhibitors cocktail 1× (Hoffmann-La Roche, Basel, Switzerland), pH 8) and subjected 5 times to 30 s of sonication on ice. Lysates were centrifuged at 5000 *g*, cell debris discarded, and soluble T domain was bound to Ni-NTA (Qiagen, Boston, MA, USA) at 4 °C for 16 h. Bound protein was washed several times with binding buffer (25 mM Tris-HCl, 300 mM NaCl, 5 mM imidazole) and eluted with 0.5 M imidazole in the binding buffer. Eluted protein was passed through a Superose 12 column 1 × 30 cm, flow rate 0.4 mL/min in 50 mM sodium-phosphate buffer, pH 8. Purified protein was quantified by absorbance at 280 nm (ε_280_ = 17.000 M^−1^cm^−1^) and purity was confirmed by SDS-PAGE.

On the request from editors to justify our mutagenesis strategy, we state that we do not believe phenylalanine to be a proper replacement for histidine, especially in case of membrane-interacting proteins, as it introduces additional non-natural mode of protein interaction with the lipid bilayer. For more discussion see [32].

Vesicle Preparation: Large unilamellar vesicles of diameter 0.1 μm were prepared by extrusion method [59,60] using a 1:3 molar mixture of POPC and POPG. Vesicles for quenching experiments contained 1:1 molar mixture of POPG and either 6-7-dibromo-PC, or 9-10-dibromo-PC, or 11-12-dibromo-PC, or POPC (control measurements with no quenching).

*T*-domain *N*-Terminus Translocation Assay: The translocation was performed using a proteolytic assay described previously [49]. Briefly, lipid vesicles composed of a molar ratio of POPG:POPC 3:1 were preloaded with ~0.02 units of bovine thrombin (Fisher Bioreagents, Fair Lawn, NJ, USA), and non-encapsulated thrombin was removed by FPLC gel filtration on a Superose 12 1 × 30 cm column. Preloaded vesicles (2 mM lipid concentration) were then mixed with 0.1 μM of *T*-domain containing an *N*-terminal His-tag linked by a sequence containing thrombin cleavage site. After 1 hour of incubation at pH 5.8, the vesicles were treated with 5% of SDS and samples analyzed by SDS-PAGE to quantify cleaved and uncleaved protein. To prevent cleavage due to vesicle rupture (rather than translocation of the *N*-terminus), 0.02 units of thrombin inhibitor hirudin (Sigma, St. Louis, MO, USA) were added to the reaction mixture.

Protein Synthesis Inhibition Assay. This assay of physiological activity of diphtheria toxin utilizes a weakened strain of full-length diphtheria toxin carrying the E148S mutation, to reduce the toxic potency [61]) (as per NIH guidelines). Full-length toxin was expressed and purified as previously detailed [62]. Inhibition of protein synthesis was determined according to the previously described method [58]. Briefly, CHO-K1 cells (10,000 cells/well) were intoxicated by diphtheria toxin constructs for 24 h at 37 °C, after which incorporation of L-[4,5-^3^H] leucine was measured.

Fluorescence Measurements: Fluorescence was measured using a SPEX Fluorolog FL 3-22 steady-state fluorescence spectrometer (Jobin Yvon, Edison, NJ, USA) equipped with double-grating excitation and emission monochromators. The measurements were made at 25 °C in 2 × 10 mm cuvettes oriented perpendicular to the excitation beam. For tryptophan fluorescence measurement, excitation wavelength was 280 nm and emission spectra was recorded between 290 nm and 500 nm using excitation and emission spectral slits of 2 and 4 nm, respectively. Normally stock concentrations of vesicles and protein were mixed to achieve final concentration of 1 μM *T*-domain and 1 mM lipid in 10 mM phosphate buffer, pH 7. The insertion was achieved by rapid addition of small aliquots of 2.5 M sodium acetate-acetic buffer, to reach the desired pH. All spectra were recorded after 30 min incubation to ensure the equilibration of the sample. Correction for the background and fitting to a log-normal distribution to determine the position of spectral maximum of emission λ_max_ was performed as described previously [63]. The spectral data were averaged to whole nm value for the presentation.

Depth-Dependent Fluorescence Quenching with Brominated Lipids: Quenching study is performed by taking a series of fluorescence measurements of the *T*-domain inserted into the vesicles containing lipids with bromine atoms attached at different positions [51,52]. Here we have used 1:1 mixture of POPG and BRPCs (6-7-dibromo-PC; 9-10-dibromo-PC and 11-12-dibromo-PC; one at a time) to prepare vesicles containing 50% quenching lipid. We have determined fluorescence intensity of tryptophan F(h) as a function of the known depth of the bromolipids (h). Data are usually normalized to the intensity in the absence of quenching, F_0_, measured with 1:1 POPC:POPG mixture For quantitative analysis we used Distribution Analysis method, which fits the data to the following symmetrical twin Gaussian function [52]:

F_0_/(F(h) − 1 = G(h − h_m_, σ, S) + G(h + h_m_, σ, S),

where

G(h − h_m_, σ, S) = S/(σ√2π) exp{−[(h − h_m_)/2σ]^2^} (1)

The three parameters of this above distribution are: most probable distance of the fluorescent probe from the center of bilayer (h_m_), dispersion of the transverse distribution of the probe (σ), and quenching efficiency (S). In this particular case, we fixed the value σ at 5.0 Å, which corresponds to a typical value observed in other systems, and used only two fitting parameters h_m_ and S.
